# Unraveling nitrogen uptake and metabolism: gene families, expression dynamics and functional insights in aspen (*Populus tremula*)

**DOI:** 10.1093/treephys/tpaf099

**Published:** 2025-08-11

**Authors:** Yupeng Zhang, Shruti Choudhary, Anna Renström, Mikko Luomaranta, Maxime Chantreau, Verena Fleig, Ioana Gaboreanu, Carolin Grones, Ove Nilsson, Kathryn M Robinson, Hannele Tuominen

**Affiliations:** Department of Forest Genetics and Plant Physiology, Umeå Plant Science Centre (UPSC), Swedish University of Agricultural Sciences, 90183 Umeå, Sweden; Department of Forest Genetics and Plant Physiology, Umeå Plant Science Centre (UPSC), Swedish University of Agricultural Sciences, 90183 Umeå, Sweden; Department of Forest Genetics and Plant Physiology, Umeå Plant Science Centre (UPSC), Swedish University of Agricultural Sciences, 90183 Umeå, Sweden; Department of Plant Physiology, Umeå Plant Science Centre (UPSC), Umeå University, 90187 Umeå, Sweden; Department of Forest Genetics and Plant Physiology, Umeå Plant Science Centre (UPSC), Swedish University of Agricultural Sciences, 90183 Umeå, Sweden; Department of Forest Genetics and Plant Physiology, Umeå Plant Science Centre (UPSC), Swedish University of Agricultural Sciences, 90183 Umeå, Sweden; Department of Forest Genetics and Plant Physiology, Umeå Plant Science Centre (UPSC), Swedish University of Agricultural Sciences, 90183 Umeå, Sweden; Department of Plant Physiology, Umeå Plant Science Centre (UPSC), Umeå University, 90187 Umeå, Sweden; Laboratory of Cell and Developmental Biology, Cluster of Plant Developmental Biology, Department of Plant Sciences, Wageningen University, The Netherlands; Department of Forest Genetics and Plant Physiology, Umeå Plant Science Centre (UPSC), Swedish University of Agricultural Sciences, 90183 Umeå, Sweden; Department of Plant Physiology, Umeå Plant Science Centre (UPSC), Umeå University, 90187 Umeå, Sweden; Department of Forest Genetics and Plant Physiology, Umeå Plant Science Centre (UPSC), Swedish University of Agricultural Sciences, 90183 Umeå, Sweden

**Keywords:** genetic variation, nitrogen assimilation, nitrogen reallocation, wood development

## Abstract

The influence of nitrogen on wood formation is well established. To gain insight into the underlying molecular mechanism, we first identified genes in 14 gene families that are involved in nitrogen uptake and metabolism in European aspen (*Populus tremula* L.) genome annotation. Gene expression data from a de novo RNA sequencing (RNA-seq) analysis and data available from the AspWood database (plantgenie.org) provided putative candidate genes for the uptake of nitrate, ammonium and amino acids from the xylem sap as well as their further assimilation in the secondary xylem tissues of the stem. For a population-wide analysis of the nitrogen-related genes, we utilized RNA-seq data from the cambial region of the stems of 5-year-old aspen trees, representing 99 natural aspen accessions, and compared the expression of the nitrogen-related genes to stem diameter. Novel regulatory interactions were identified in expression quantitative loci and co-expression network analyses in these data. The expression of certain nitrate and amino acid transporters correlated negatively with stem diameter, suggesting that excessive nitrogen retrieval from the xylem sap suppresses radial growth of the stem. The expression of a glutamine synthetase correlated with the expression of these transporters, a link further supported by increased plant growth in transgenic glutamine synthetase overexpressing trees. This study provides insight into the genetic basis of nitrogen uptake and assimilation and its connection to wood formation, providing interesting targets for improving nitrogen-use efficiency and growth of aspen trees.

## Introduction

Nitrogen is an essential macronutrient for plant growth and development, playing a critical role in a variety of physiological and biochemical processes, including photosynthesis, amino acid and protein synthesis, metabolic regulation, growth and biomass production. Nitrogen availability also influences wood formation, either directly or indirectly, in many ways (for a recent review, see Lu et al. 2024). In *Populus* trees, high nitrogen availability has been reported to increase vessel and fiber lumen area and to reduce secondary cell wall thickness, lignin content and wood density (Luo et al. 2005, Pitre et al. 2007, Novaes et al. 2009, Cao et al. 2024, Renström et al. 2024).

Nitrogen-use efficiency, which refers to the ability of a plant to acquire and utilize nitrogen to maximize growth and yield, is a key aspect of modern agriculture and forestry (Congreves et al. 2021, Q.Liu et al. 2022). Increased understanding of the molecular mechanisms that underpin nitrogen uptake, transport and metabolism can provide candidate genes to increase nitrogen-use efficiency of plants and to improve sustainability and minimize environmental impacts caused by excessive use of nitrogen fertilizers (Cánovas et al. 2018, Stevens 2019, Waqas et al. 2023). While significant progress has been made in studying nitrogen-related pathways in model plants like *Arabidopsis thaliana* (Arabidopsis), there is still much to learn about how these mechanisms operate in long-lived woody species. Recent advances in genomics and transcriptomics have enabled investigation of the genes and pathways involved in nitrogen metabolism in various plant species, including woody species. In black cottonwood (*P. trichocarpa*), nitrogen-related gene families, such as amino acid permeases (AAPs), nitrate transporters (NRTs), nitrite reductases (NIRs) and NIN-like proteins (NLPs) have been identified (Couturier et al. 2010, Bai et al. 2013, Léran et al. 2014, von Wittgenstein et al. 2014, Wu et al. 2015, Xu et al. 2017, Du et al. 2022, Han et al. 2022, Cao et al. 2023, Yu et al. 2023, Li et al. 2024, Guan et al. 2025). These gene families often exhibit substantial variation in gene number, structure and function across plant species, reflecting evolutionary pressures and ecological adaptations. However, the extent of this variation remains poorly characterized in other *Populus* species.

In this study, we investigated nitrogen-related gene families in European aspen (aspen, *Populus tremula*), which is the only native *Populus* species in Sweden and extensively used as a deciduous tree model for population genetics and molecular studies (Escamez et al. 2023, Luomaranta et al. 2024, Robinson et al. 2024). We identified the members of the following gene families: amino acid transporters (AAT), ammonium transporters (AMT), asparagine synthetases (ASN), asparaginases (ASPG), alanine aminotransferases (AlaAT), aspartate aminotransferases (AspAT), cationic amino acid transporters (CAT), glutamate dehydrogenases (GDH), glutamate synthases, glutamine synthetases (GS), NIR, nitrate reductases (NR), NLP and NRT in aspen, and constructed phylogenetic trees including genes from aspen and Arabidopsis. We also revealed tissue specific expression patterns and specific responses to nitrate vs ammonium in developing xylem tissues of hybrid aspen trees. Additionally, expression quantitative trait loci (eQTL) mapping and gene co-expression networks highlighted links between nitrogen metabolism and radial growth of the stem.

## Materials and methods

### Gene family identification in aspen

*Arabidopsis thaliana* (Arabidopsis) genes from 14 gene families encoding AAT, AMT, ASN, ASPG, AlaAT, AspAT, CAT, GDH, glutamate synthases (GOGAT), GS, NIR, NR, NIN-like transcription factors (NLP) and NRT were extracted from The Arabidopsis Information Resource (TAIR; https://www.arabidopsis.org). These sequences were utilized as queries for a BLASTP search against the *Populus tremula* v2.2 genome annotation on plantgenie.org by using default settings (Sundell et al. 2015, Robinson et al. 2024). As a second step, the nitrogen-related genes from Arabidopsis were analyzed for conserved functional motifs searching the Pfam database via the HMMER v3.4 tool (hmmscan, Potter et al. 2018). The identified motifs served as seed motifs for HMMER (hmmsearch, Potter et al. 2018) against the annotated peptide sequences in *Populus tremula* v2.2, resulting in an initial gene list. Genes from the first BLASTP output that, on the basis of the second screening, lacked the conserved functional motifs were excluded from the final list of nitrogen-related genes in aspen. The aspen genes were named after Arabidopsis genes with the highest sequence similarity according to BLASTP.

### Phylogenetic tree construction and the analyses of the gene structures and conserved domains

The peptide sequences of identified genes were aligned using default settings of MAFFT v7.526 (Katoh and Standley 2013). The aligned sequences were trimmed by trimal v 1.5.rev0 with default settings (Capella-Gutiérrez et al. 2009). The aligned sequences were then employed to construct a maximum likelihood phylogenetic tree with 1000 bootstrap replicates using IQ-TREE2 v2.3.6 (Minh et al. 2020). The resulting phylogenetic trees were visualized with MEGA11 (Tamura et al. 2021). The identified motifs from hmmerscan and the structure of the genes were visualized by TBTOOL2 (Chen et al. 2023).

### Gene expression in the AspWood database

The Aspen Wood (AspWood) resource of high-resolution gene expression data from the developing wood of aspen trees (Sundell et al. 2017) were downloaded from PlantGenIE (https://plantgenie.org, Sundell et al. 2015). The expression data were presented as log2(TPM + 1) values and visualized as heatmap using ComplexHeatmap v2.20.0 (Sundell et al. 2017, Gu 2022).

### RNA-sequencing of ammonium treated hybrid aspen trees

The experiment is described in full in Renström et al. (2024). Briefly, hybrid aspen (*P. tremula* L. × *P. tremuloides* Michx, clone ‘T89’) trees were cultivated in greenhouse conditions for 10 weeks under controlled nutrient additions. Trees were fertilized with ammonium-based nutrient solution at three different addition levels: limited, sub-optimal and optimal, corresponding to total nitrogen amounts of 0.16, 0.56 and 1.06 g, respectively. At the end of the experiment, 10-cm-long stem pieces were collected from the base of the plant and immediately frozen in liquid nitrogen. The developing xylem part of the stem piece was then scraped off and homogenized using a mortar and pestle in liquid nitrogen. RNA was extracted from the homogenized material using the Spectrum™ Plant Total RNA Kit (Sigma-Aldrich Co. LLC). RNA was quantified with a Nanodrop 1000 Spectrophotometer (Thermo Scientific), and the quality was assessed with an Agilent 2100 Bioanalyzer (Agilent Technologies, Santa Clara, CA, USA). Following sequencing library generation and paired-end (2 × 150 bp) sequencing using Illumina NovaSeq 6000, the raw reads were pre-processed to remove sequencing adapters using Trimmomatic (v0.39). The trimmed read pairs were quantified with Salmon (v1.9) using the transcriptome index based on aspen genome annotation (Robinson et al. 2024). For exploratory analysis, including sample clustering and principle component analysis (PCA) visualization, we applied variance stabilizing transformation (VST) to the normalized counts using DESeq2’s vst() function. Formal differential expression testing was subsequently performed on raw counts using DESeq2 (v1.46.0 in R 4.4.2), which internally handles count normalization via median-of-ratios scaling (Love et al. 2014). Statistical significance between the RNA-Seq results for the ammonium-treated (analyzed here) and nitrate-treated trees (published in Renström et al. 2024) was tested using Tukey’s Honestly Significant Difference (HSD) post-hoc tests on the normalized expression values. The cld() function (multcompView v0.1–10) generated the compact letter display, indicating significant differences (adjusted *P* < 0.01) between all treatment combinations (3 concentrations × 2 nitrogen treatments).

### Expression quantitative trait loci analysis in the Swedish aspen collection

The eQTL data for the Swedish aspen (SwAsp) trees were retrieved from Luomaranta et al. (2024). Briefly, the eQTL analysis was based on RNA-seq analysis of developing xylem tissue collected from 5-year-old stems, representing 99 SwAsp genotypes. The eQTL analysis was based on 6,806,717 bi-allelic SNPs (Robinson et al. 2024). Mean normalized gene expression values were used for each genotype. The false discovery rate threshold of 0.05 was applied to exclude non-significant eQTLs. eQTLs were categorized as local (within 1 Mbp) or distant.

### The subnetwork of nitrogen-related genes

The whole-transcriptome co-expression network from Luomaranta et al. (2024) was used to extract a sub-network of nitrogen-related genes using Cytoscape v3.10.2 (Shannon et al. 2003). The sub-network was clustered by using INFOMAP (v.1.8.0; Rosvall and Bergstrom 2008). Stem diameter of the 99 different SwAsp genotypes (Luomaranta et al. 2024) was added as a node in the sub-network. Correlation of stem diameter to each gene in the sub-network was then represented according to the calculated Spearman rank correlation (*R* > 0.3) with only edges.

### Overexpression of the *Populus* glutamine synthetase *GLN1.2a* and the growth of the transgenic lines

A DNA fragment corresponding to the coding sequence of *Populus tremula PotraGLN1.2a* (Potra2n12c24087), flanked by the Gateway attL1 and attL2 cloning sequences, was synthesized and inserted into pUC57 by the GenScript company. The resulting vector was recombined in an LR reaction with the destination vector pK2GW7. The destination vector, directing the expression of *PotraGLN1.2a* under the control of the 35S promoter, was transformed into hybrid aspen (*Populus tremula* × *P. tremuloides*), as described in Nilsson et al. (1992).

The transgenic trees were grown in an automated phenotyping platform (WIWAM Conveyor, Eeklo, Belgium) for 7.5 weeks under long-day conditions (18 h/6 h day/night) with 160–230 μmol m^−2^ s^−1^ light intensity from white light (FL300 LED Sunlight v1.1) and far red light (FL100 LED custom-made, 725–735 nm) lamps (Senmatic A/S, Søndersø, Denmark), 22 °C/18 °C temperature regime, and 60% relative humidity, as described in Wang et al. (2022). The expression of the transgene was analyzed in in vitro material (whole shoots) from tissue culture by quantitative reverse transcription polymerase chain reaction (qPCR). Briefly, 1 μg RNA of at least three biological replicates was taken for cDNA synthesis using the iScript™ cDNA Synthesis Kit (Bio-Rad, USA). Primers were designed using the Primer3 web server (v.4.1.0; Untergasser et al. 2012). Forward and reverse sequences, for the *PotraGLN1.2a* (Potra2n12c24087) were GTCTGACTGGTCGCCATGAA and AGCTTTCTCTGTGTCCCTGC, respectively, with ubiquitin (Potra2n1c3635; 5′-AGATGTGCTGTTCATGTTGTCC-3′, 5′-ACAGCCACTCCAAACAGTACC-3′) as a reference. qPCR was performed in 10 μL reaction volume, comprising of 1 μL of 5× diluted cDNA, 1× PowerTrack™ SYBR Green Master Mix (Thermo Fisher Scientific), 100–200 nmol each of forward and reverse primer. All reactions were performed in 96-well plates on a C1000Touch™ Thermal Cycler (Bio-Rad) with an initial denaturation at 95 °C for 3 min, followed by 40 cycles, each of denaturation at 95 °C for 10s, and annealing at 60 °C for 10 s. The *C*_q_ values were acquired from the CFX96™ Maestro software (Bio-Rad) and average dCq shown for each sample were calculated relative to ubiquitin.

## Results

### Identification, phylogeny and expression analysis of nitrogen uptake and metabolism related gene families in aspen

**Figure 1:**
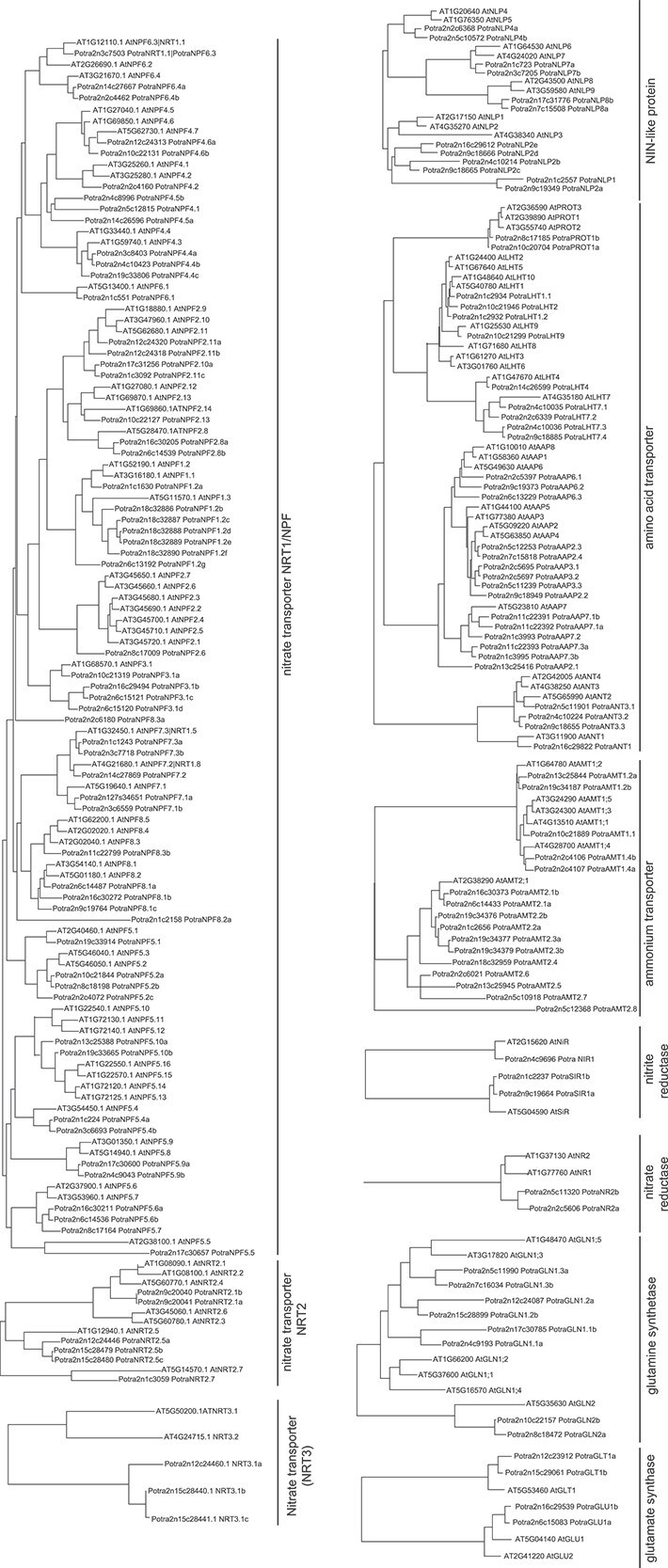
Identification of nitrogen uptake and metabolism-related genes in aspen (*P. tremula*). Phylogenetic trees include genes encoding members of the nitrate transporter families, NIN-like transcription factors, AAT, AMT, glutamate synthases, glutamine synthetases, NIR and NR in aspen and Arabidopsis. The peptide sequences of genes in corresponding gene families were aligned by MAFFT. The maximum likelihood tree with 1000 bootstraps was constructed by IQTREE2 and visualized by MEGA11.

**Figure 2:**
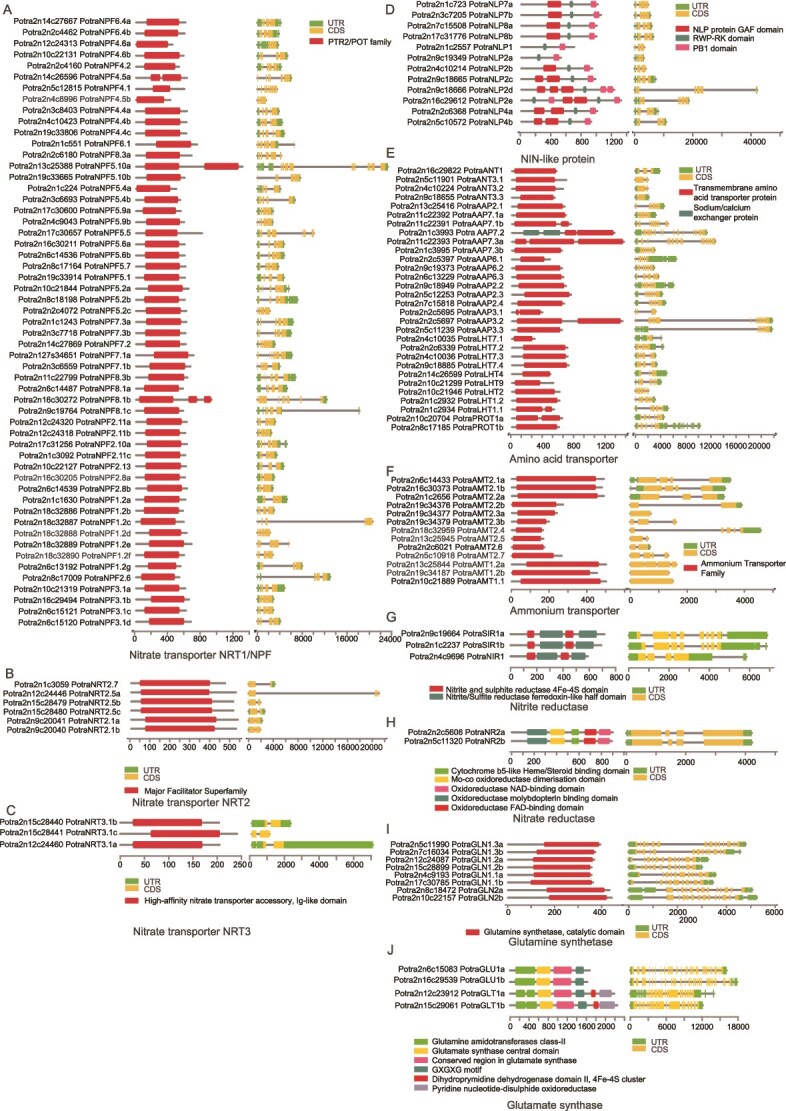
Gene structure and conserved protein motifs of nitrogen uptake and metabolism-related gene families in aspen. Distribution and the relative genomic positions of conserved motifs, identified by hmmscan, are shown for nitrate transporter (A, B, C), NIN-like protein (D), amino acid transporter (E), ammonium transporter (F), nitrite reductase (G), nitrate reductase (H), glutamine synthetase (I) and glutamate synthase (J) gene families. Exons are shown as yellow boxes, introns as black lines and untranslated regions as green boxes. Gene lengths are indicated at the x axis relative to the transcription start site (0 bp).

In order to facilitate molecular analyses underlying nitrogen-mediated responses in wood formation, we first identified the aspen members of 14 gene families related to nitrogen uptake and metabolism (Figure 1, Table S1 available as Supplementary Data at *Tree Physiology* Online). Phylogenetic trees containing aspen and Arabidopsis were constructed to provide insight into the evolutionary relationship (Figure 1). In addition, the overall gene structure was analyzed for all genes (Figure 2).

**Figure 3:**
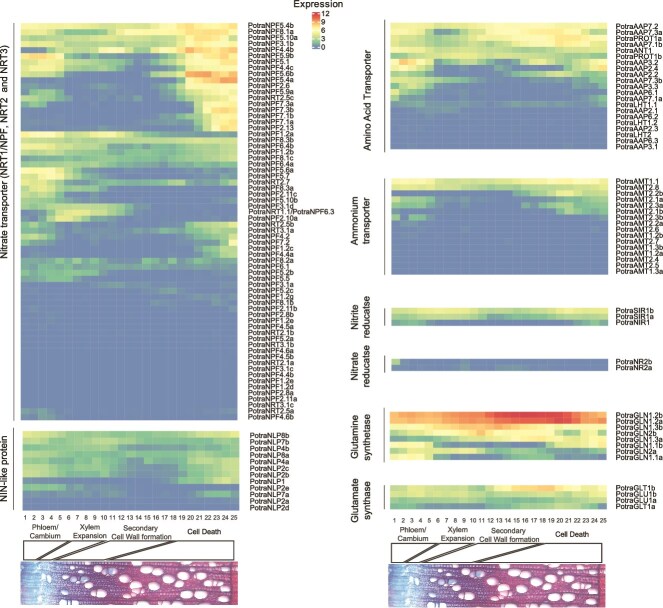
The expression of the nitrogen uptake and metabolism-related genes in aspen. The heatmap represents log(TPM + 1) values for the different genes in the AspWood database (plantgenie.org). Horizonal axis indicates the identity of the samples (in the AspWood tree 1) and the different zones of the cambial region (the phloem, the phase of xylem expansion, the phase of secondary cell wall formation and cell death) in aspen stem. The cambium (˃85% of the cell population) is in samples 3–6 and the expanding xylem (>85% of the cells) is in samples 7–11. According to Sundell et al. (2017), the vessels die and autolyze around the sample number 18 while the fibers are dead only in the last sample where the only living cells are the rays in tree 1 of the AspWood database. The colors indicate relative expression levels according to the scale on the right.

We also used the publicly available AspWood dataset to extract information of gene expression in the woody tissues of aspen for the nitrogen-related genes identified in this study. AspWood is a high-resolution gene expression database in the woody tissues of aspen stems (Sundell et al. 2017), including data from the phloem/cambium region, and the different phases of xylem expansion, xylem maturation and xylem cell death (plantgenie.org; Sundell et al. 2015). Several members of the nitrogen-related gene families were according to the AspWood database expressed in a biphasic manner in aspen, with a first peak in the phloem/cambium and a second peak in the phase of xylem cell death (Figure 3). The expression in the phloem/cambium is likely related to the transport and sensing of nitrogen that is being transported in the phloem, while the expression in the phase of cell death is most likely related to the transport and sensing of nitrogen compounds that have been taken up from the xylem sap to ray parenchyma (van Bel 1984, Tegeder 2014, Cánovas et al. 2018).

### NIN-like proteins

The NIN-like transcription factors (NLPs) initiate nitrate signaling and coordinate gene expression related to nitrate transport and metabolism, and plant development (Vidal et al. 2020). Arabidopsis has nine *NLP* genes, and the AtNLP7 has been shown to both directly bind nitrate and act as a transcription factor (K.H.Liu et al. 2022). We identified 12 *NLP* genes in aspen (Figures 1 and 2, Table S1A available as Supplementary Data at *Tree Physiology* Online), most of them having the biphasic expression pattern in woody tissues (Figure 3). The aspen *PotraNLP7a*, homologous to the Arabidopsis *AtNLP7,* was expressed in phloem/cambial tissues and during xylem expansion. The highest expressed gene was *PotraNLP8b,* which has the highest sequence similarity to the Arabidopsis *AtNLP8* and *AtNLP9* (Figure 1).

### Nitrate transporters

Nitrate transporters are vital for the uptake and partitioning of nitrate in plants. They can be found in several different families, including the Nitrate transporter 1/Peptide transporter Family (NRT1/NPF) (Léran et al. 2014), the Nitrate Transporter 2 (NRT2) (von Wittgenstein et al. 2014) and Nitrate Transporter 3 (NRT3) (Wang et al. 2018) families. The NRT1/NPF family members are typically so-called low-affinity transporters (Corratgé-Faillie and Lacombe 2017) that are active at nitrate concentrations exceeding 250 μM, while members of the NRT2 family seem to represent high-affinity transporters that are generally active at nitrate concentrations in the range of 10–250 μM (Xu et al. 2024). The first described nitrate transporter, the Arabidopsis AtNRT1.1 (also called CHL1 or NPF6.3), is an exception in that it can operate as a dual affinity transporter (Liu et al. 1999). NRT3 is necessary for NRT2 stability and targeting to the plasma membrane (Wang et al. 2018).

The NRT1/NPF gene family was one of the largest families in aspen. We found 57 aspen genes corresponding to the 53 Arabidopsis genes (Figures 1 and 2, Table S1B available as Supplementary Data at *Tree Physiology* Online). The aspen NRT2 gene family contained six aspen genes corresponding to the seven genes in Arabidopsis while the NRT3 family had three aspen genes corresponding to the two Arabidopsis genes (Figures 1 and 2, Table S1B available as Supplementary Data at *Tree Physiology* Online).

A large proportion of the genes in the aspen nitrate transporter gene families were expressed in the woody tissues (Figure 3). Several of them showed the biphasic expression pattern (Figure 3). Examples of such genes included *PotraNPF4.4c* and *PotraNPF7.3a*, homologs of which are well characterized in Arabidopsis, as well as *PotraNPF5.4a*, *PotraNPF5.4b* and *PotraNPF5.6b,* which have not been functionally characterized as nitrate transporters in Arabidopsis. *PotraNRT1.1/NPF6.3* had a distinct expression pattern within the family: its expression was not biphasic but peaked in the zone of xylem cell expansion (Figure 3). The two aspen homologs of the high-affinity *AtNRT2.1* both had very low expression in the woody tissues (Figure 3).

### Ammonium transporters

AMTs mediate the uptake of ammonium from the soil, but also ammonium transport from root to shoot and within leaves, and ammonium acquisition into other organs (Hao et al. 2020). The *AMT* genes in Arabidopsis are divided in two phylogenetic groups; one containing five genes (*AtAMT1;1*—*AtAMT1;5*) and the other one containing the single *AtAMT2;1*. Both AMT1 and AMT2 show high affinity to ammonium (Yuan et al. 2007).

The AMT gene family showed clear clustering into two main clades (Figure 1, Table S1C available as Supplementary Data at *Tree Physiology* Online). The clade corresponding to Arabidopsis *AtAMT1;1–1;5* contained five aspen genes. *PotraAMT1.1* showed a strong, biphasic expression in the woody tissues (Figure 3). The second clade, containing the Arabidopsis *AtAMT2;1*, has undergone copy number expansion with 11 aspen genes (Figure 1, Table S1C available as Supplementary Data at *Tree Physiology* Online). Several of the aspen *AMT2* clade members were expressed at a low level primarily in the cell death zone except for *PotraAMT2*.*8* that had a constant, low expression in the woody tissues (Figure 3).

### Amino acid transporters

Transport of amino acids and other nitrogenous compounds in plants is primarily mediated by two major superfamilies: the amino acid-polyamine-organocation (APC) superfamily and the amino acid/auxin permease (AAAP) superfamily (Pratelli and Pilot 2014). We focused on four functionally characterized subfamilies within these superfamilies: the AAP and the lysine/histidine transporters (LHT) from the AAAP superfamily, and the proline transporters (ProT) and the aromatic and neutral amino acid transporters (ANT) from the APC superfamily (Tegeder and Hammes 2018, Yang et al. 2020). Aspen contained 15 *AAP*, 9 *LHT*, 2 *ProT* and 4 *ANT* family members (Figures 1 and 2, Table S1D available as Supplementary Data at *Tree Physiology* Online). They were expressed primarily in the phloem/cambium (*PotraANT1, PotraAAP3.2, PotraAAP3.3, PotraAAP6.1, PotraAAP7.3b*), the expansion zone (*PotraPROT1b*), secondary cell wall formation (*PotraAAP7.2*, *PotraAAP7.3a*) and the cell death zone (*PotraProT1a*, *PotraAAP*7.1b) (Figure 3).

### Nitrogen assimilation and metabolism

Nitrate is reduced to nitrite by the cytoplasmic enzyme nitrate reductase (NR). Two *NR* genes, clustering together with the Arabidopsis *AtNR1* (also known as *NIA1*) and *AtNR2* (also known as *NIA2*), were annotated in the aspen genome (Figures 1 and 2, Table S1F available as Supplementary Data at *Tree Physiology* Online). *PotraNR2a* and *PotraNR2b* had a low expression in the woody tissues (Figure 3).

Nitrite is subsequently imported into plastids, where nitrite reductase (NIR) reduces it to ammonium. One aspen homolog was found for the Arabidopsis nitrite reductase *AtNIR1* while two homologs were found for a sulfite reductase (*AtSIR*, AT5G04590) which both contained a Nitrite/Sulfite reductase ferredoxin-like domain (Figures 1 and 2, Table S1E available as Supplementary Data at *Tree Physiology* Online). *PotraNIR1* had a low, biphasic expression in the phloem/cambium and cell death zone while *PotraSIR1a* and *PotraSIR1b* had a broader and higher expression, including expression in the xylem expansion zone (Figure 3).

Ammonium is assimilated in both the cytosol and plastids to produce amino acids in a set of interconnected reactions. In the cytosol, ammonium can react with glutamate to produce glutamine by the cytosolic glutamine synthetase (GS) or asparagine by asparagine synthetase (ASN). In the plastids, a plastidic GS catalyses conversion of ammonium to glutamine, which can be then converted to glutamate by glutamate synthase (GOGAT) in the so-called GS/GOGAT cycle. Asparaginase (ASPG), alanine aminotransferase (AlaAT), aspartate aminotransferase (AspAT) and glutamate dehydrogenase (GDH) are additional enzymes involved in nitrogen assimilation.

In the aspen genome, we identified eight glutamine synthetase, four glutamate synthase, three asparagine synthetase, four glutamate dehydrogenase, three asparaginase, two alanine aminotransferase and nine aspartate aminotransferase genes (Figures 1 and 2, Table S1G–N available as Supplementary Data at *Tree Physiology* Online). Each of these gene families had at least one gene that was highly expressed in the woody tissues (Figure 3). A few members of both the glutamate synthase (*PotraGLT1b*) and the glutamine synthetase (*PotraGLN1.2a* and *PotraGLN1.2b*) families had a constant, high expression throughout wood development (Figure 3).

### The expression of the nitrogen uptake and metabolism-related genes under NH₄^+^ and NO₃^−^ treatment

*Populus* species can take up nitrogen in the forms of both ammonium (NH₄^+^) and nitrate (NO₃^−^), but the site of assimilation and hence the transported form of nitrogen in the stem varies depending on the genotype and the environmental conditions (Black et al. 2002). In this study, we aimed to shed light on nitrate assimilation and subsequent nitrogen metabolism by exploring the effect of both NH₄^+^ and NO₃^−^ on the expression of the nitrogen-related genes in developing xylem tissues of wood. For this purpose, we utilized material and data from our earlier study (Renström et al. 2024) where hybrid aspen trees were grown for 2 months in three different levels (suboptimal, low and optimal) of either NH₄^+^ or NO₃^−^. The nitrogen levels were defined in Renström et al. (2024) on the basis of known amounts of total nitrogen present in trees reaching maximal growth rates. The RNA-seq data in the nitrate-fertilized trees were retrieved from Renström et al. (2024) while the RNA-seq experiments in the ammonium-fertilized trees were performed in this study (see Table S2 available as Supplementary Data at *Tree Physiology* Online).

**Figure 4:**
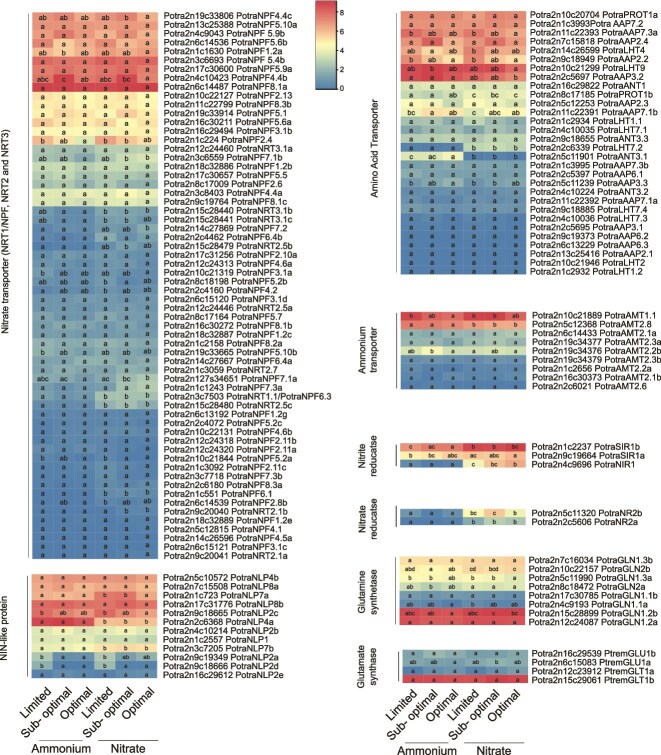
The expression of the members of aspen gene families related to nitrogen uptake, sensing and assimilation in response to fertilization with either nitrate or ammonium. The heatmaps represent VST normalized gene expression data from RNA-sequencing of developing xylem tissues of hybrid aspen after long-term treatment with optimal, sub-optimal and limited doses of ammonia (NH_4_^+^, this study) and nitrate (NO_3_^−^, reported earlier in Renström et al. 2024). Different letters indicate statistically significant differences using two-way ANOVA model and Tukey’s HSD test with adjusted *P*-value < 0.01. The number of the trees in each condition is three or four.

Almost all members of the nitrate transporter gene families were expressed in at least one of the experimental conditions (Figure 4). Half of them were expressed at a higher level when plants were fertilized with nitrate compared with ammonium (see Figure S1 available as Supplementary Data at *Tree Physiology* Online) even though statistically significant differences were present only for a few of them, including *PotraNRT1.1/NPF6.3*, *PotraNRT2.5c* and *PotraNRT2.1b* (Figure 4). Also nitrate and nitrite reductases as well as the two homologs of the *AtNLP7* nitrate sensors (*PotraNLP7a* and *PotraNLP7b*) were significantly more expressed in response to nitrate than ammonium (Figure 4). The AMT family members were rather equally expressed even though tendencies toward higher expression of a few of them were observed in response to fertilization with nitrate (Figure 4). The amino acid transporter families contained specifically responding genes but also genes that responded similarly to both nitrogen sources (Figure 4).

### Expression quantitative trait loci analysis of nitrogen-related gene expression in the Swedish aspen collection

Members of the NLP gene family are interesting since they function in sensing of nitrate but also as transcription factors. We were interested in the regulatory aspects of this gene family, and utilized eQTL data from a population of aspen trees (the SwAsp collection) to investigate variation in the expression and regulation of the *NLP* genes in woody tissues (Luomaranta et al. 2024). The eQTLs were classified either as local or distant using 1 Mbp as a threshold (Luomaranta et al. 2024). The different members of the NLP gene family contained 333 local eQTLs and 336 distant eQTLs, corresponding to association with 28 and 179 genes, respectively (see Table S3 available as Supplementary Data at *Tree Physiology* Online). The SNPs in *PotraNLP2e* showed association with the expression of 34 different genes, including homologs of Arabidopsis *GA 20-oxidase* and *UMAMIT34* as distant eQTLs and *PotraGLU1b* as a local eQTL (see Table S3 available as Supplementary Data at *Tree Physiology* Online). Distant eQTLs for *PotraNLP7a* and *PotraNLP7b* were found in association with the expression of 12 and 13 genes, respectively, several of them having predicted functions in signaling and transcriptional regulation (see Table S3 available as Supplementary Data at *Tree Physiology* Online).

To broaden the scope of the expression analysis, we retrieved co-expression data for the nitrogen-related genes from a whole-transcriptome co-expression analysis in woody tissues of young aspen stems from Luomaranta et al. (2024), and constructed a subnetwork with these values together with the Pearson correlation values for the expression of these genes with the stem diameter of the same set of trees that were used for the RNA-Seq analysis.

**Figure 5:**
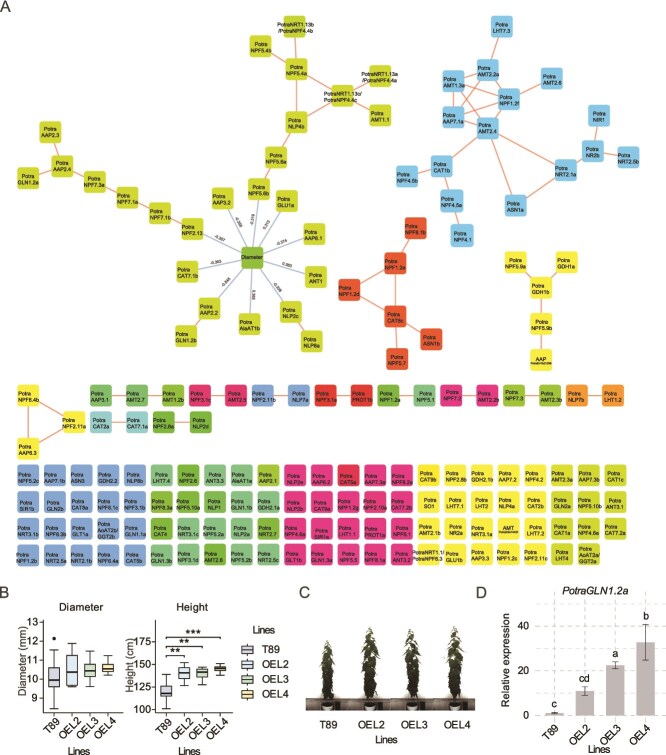
Co-expression of the nitrogen uptake and metabolism-related aspen genes in stem tissues of a population of SwAsp trees. (A) A subnetwork was calculated by extracting the co-expression values for the aspen nitrogen-related genes from a whole-transcriptome network, published in Luomaranta et al. (2024), that was based on gene expression in developing xylem tissues of 5-year-old aspen trees representing 99 genotypes of the SwAsp population. Correlation between genes in the Seidr whole-transcriptome network are indicated by orange lines. The Spearman correlation values (*R* > 0.3) between the diameter data of the stems and gene expression were included as edges, indicated as blue lines in the figure. (B) Phenotypic characterization of glutamine synthetase *PotraGLN1.2a* overexpression lines in hybrid aspen. Data for stem diameter and height of the trees is shown for the wild type (T89) and three *PotraGLN1.2a* overexpression lines grown for 2 months in an automated phenotyping platform. (C) Photos of representative trees in each of the wild type (T89) and transgenic *PotraGLN1.2a* overexpressor lines at the end of the growth period. (D) The relative expression of *PotraGLN1.2a* in in vitro tissues of wild type (T89) and three transgenic *PotraGLN1.2a* overexpressing lines (L2, L3, L4). Different letters indicate statistically significant differences using two-way ANOVA model and Tukey’s HSD test with adjusted *P*-value < 0.01. The number of trees in each condition is three.

A large proportion of the genes (91) were not co-expressed at all, a few genes were co-expressed with only one or two genes, and 54 genes were found in four clusters containing more than three co-expressed genes (Figure 5A). The largest cluster contained 26 genes, including the NPF family members *PotraNPF5.4a*, *PotraNPF5.4b* and *PotraNPF5.6b* that were also highly expressed in the woody tissues according to the AspWood database (Figure 3). This cluster also contained several AAP family members, such as *PotraAAP2.2*, *PotraAAP3.2*, *PotraAAP6.1* and *PotraANT1*, and the NRT1/NPF family members *PotraNPF5.6b* and *PotraNPF2.13*, which all correlated negatively with stem diameter, and glutamine synthetases *PotraGLN1.2a* and *PotraGLN1.2b* (Figure 5A).

To gain more understanding on the significance of the co-expression patterns in the largest cluster, we analyzed the effect of *PotraGLN1.2a* overexpression on tree growth in greenhouse conditions. *PotraGLN1.2a* was selected due to its high expression in the secondary xylem tissues (Figure 3). Its expression is also specific to wood (plantgenie.org). Three hybrid aspen (*Populus tremula* × *P. tremuloides*) overexpression lines of *PotraGLN1.2a* were grown in controlled conditions. After 2 months of growth, the *PotraGLN1.2a* overexpression lines were significantly taller than the wild-type trees (Figure 5B and C). Positive influence of *GS* overexpression on the growth of *Populus* trees has also been reported in several earlier studies (Gallardo et al. 1999, Fu et al. 2003, Jing et al. 2004, Castro-Rodríguez et al. 2016). Altogether, these results suggest that while tree growth is positively influenced by the expression of glutamine synthetases, it is counteracted by the expression of AAP and NRT1/NPF family members in the secondary xylem tissues of the stem.

## Discussion

### Identification of nitrogen uptake and metabolism related gene families in aspen

Here, we performed identification and analysis of 14 nitrogen uptake and metabolism associated gene families in aspen. Even though the function of many of these genes is known in Arabidopsis, little is known about their role in woody tissues of trees. Our analyses did not always identify the best characterized members of the gene families as those that seemed most important in aspen stem based on gene expression. For instance, several *NRT1/NPF* genes, such as *PotraNPF5.4*a, *PotraNPF5.4b* and *PotraNPF5.6b* which were highly expressed in the woody tissues of aspen stem (Figure 3), have not been established as nitrate transporters in any other species. Their role in nitrate sensing or transport is supported by their co-expression in the population of the SwAsp trees (Figure 5A), and in particular their co-expression with *PotraNPF4.4* which is a homolog of the Arabidopsis nitrate-binding protein *AtNPF4.4/NRT1.13* (Chen et al. 2021). The protein sequences of PotraNPF5.4a, PotraNPF5.4b and PotraNPF5.6b also contain the proline residue that is required for nitrate transport activity (see Figure S2 available as Supplementary Data at *Tree Physiology* Online; Ho et al. 2009, Chen et al. 2021). We therefore propose involvement of these aspen *NPF* genes in nitrate uptake and/or sensing in secondary xylem tissues of the stem. Recently, a cassava homolog of *PotraNPF5.4* was shown to be specifically expressed in the stem and decrease the efflux of nitrate in the root when overexpressed in rice, supporting the role of NPF5.4 in nitrate uptake from the xylem sap (Ji et al. 2024).

The highest expressed *NLP* gene in aspen wood was *PotraNLP8b* (Figure 3)*,* which is most similar in sequence to Arabidopsis *AtNLP8* and *AtNLP9*. *AtNLP8* and *AtNLP9* are not very well characterized in Arabidopsis (Konishi et al. 2021). *AtNLP8* gene has been reported to be expressed in imbibed seeds and to promote seed germination (Yan et al. 2016). *AtNLP9* is expressed, similar to *PotraNLP8*, in the seeds, but also in procambium of the root (Brady et al. 2007). Two *Populus NLP8* homologs were recently mapped to QTL loci associated with tree biomass related traits in *P. deltoides* × *P. simonii* F1 population although their direct involvement was not demonstrated (Du et al. 2023). Taken together, the homologs of *AtNLP8* and *AtNLP9* genes seem to have pivotal roles in nitrate signaling of woody tissues in *Populus* trees.

### Nitrogen uptake and assimilation in the developing xylem tissues of *Populus* stems

*Populus* trees, such as aspen, are known to be able to utilize both nitrate (NO_3_^−^) and ammonium (NH_4_^+^) even though nitrate is the predominant source of nitrogen (Rennenberg et al. 2010). We showed earlier that the growth of hybrid aspen trees was comparable when using either ammonium or nitrate as the sole nitrogen source, demonstrating that both sources can be equally utilized (Renström et al. 2024). However, it is not clear where in the tree nitrogen that is taken up in the form of nitrate is being assimilated. Preferential assimilation of nitrate in both the shoots (Black et al. 2002) and the roots (Gessler et al. 2004) have been reported. We observed that fertilization with nitrate activated the so-called primary nitrate response (Krouk et al. 2010), including induction of the expression of both nitrate and nitrite reductases, in developing xylem tissues of the stem (Figure 4). This observation demonstrates that nitrate that is being taken up by the roots is not necessarily assimilated in the roots but transported in the xylem sap of the stem and taken up in the stem where it stimulates the expression of nitrate-assimilating genes. However, nitrate treatment also tended to increase the expression of a few members of the AMT gene family (Figure 4), which could reflect regulation of these *AMT*s by nitrate or that at least a part of the applied nitrate is assimilated in the roots and transported in the form of ammonium. Furthermore, the expression of amino acid transporters in the secondary xylem tissues in response to nitrate fertilization supports the assimilation of nitrate before reaching the xylem tissues of the stem (Figure 4). It is therefore likely that nitrate can be assimilated in both the roots and the above ground parts of the trees.

The capacity of applied nitrate to induce the expression of *NR* and *NIR* in the secondary xylem tissues raises the question of how nitrate is removed from the xylem sap. The tissue-specific expression pattern and nitrate responsiveness indicated on the action of specific *NRT1/NPF* genes, such as *PotraNPF4.4b* and *PotraNPF7.2,* in the lateral transport of nitrate from the xylem sap to the surrounding parenchymatic cells (Figures 3 and 4). *PotraNPF4.4b* is homologous to the Arabidopsis *AtNRT1.13/NPF4.4* which is expressed in the parenchymatic cells next to the xylem elements, but which cannot transport nitrate from the sap since it lacks the proline residue crucial for nitrate transport (Chen et al. 2021). Interestingly, PotraNPF4.4b contains the proline residue (see Figure S2 available as Supplementary Data at *Tree Physiology* Online), supporting its function in nitrate uptake from the xylem sap. *PotraNPF7.2* is a promising candidate for having a role in nitrate retrieval from the sap to the xylem parenchyma on the basis of its strictly ray cell specific expression (Figure 3 and Figure S3 available as Supplementary Data at *Tree Physiology* Online, Tung et al. 2023) and homology to *AtNRT1.8/NPF7.2* which is expressed in xylem parenchyma cells and supposedly removes nitrate from the xylem vessels (Li et al. 2010). Additionally, the aspen homologs of *AtNRT1.5/NPF7.3* which participates in nitrate reallocation in Arabidopsis (Lin et al. 2008) could play a role in nitrate reallocation in tree stems.

### Assimilation of nitrogen and radial growth of tree stems

Xylem-to-phloem allocation of amino acids has been demonstrated in lupin (Pate et al. 1975) and tomato (van Bel 1984). We identified several members of the amino acid transporter families that were highly expressed in the developing xylem tissues of aspen trees (Figure 3) and that could therefore have a function in the uptake of amino acids from the xylem sap into the parenchymatic ray cells of the secondary xylem. Interestingly, the results from the co-expression analysis in the SwAsp population of aspen trees showed that the expression of three AAPs, *PotraAAP2.2, PotraAAP3.2* and *PotraAAP6.1*, correlated negatively with stem diameter (Figure 5A). *PotraAAP2.2* is homologous to *AtAAP2* (AT5G09220) which mediates root-derived amino acid transport from xylem to phloem (Zhang et al. 2010) and coordinates partitioning of nitrogen and carbon within the plants (Perchlik and Tegeder 2018). The loss of AtAAP2 function in Arabidopsis resulted in increased allocation of nitrogen into the leaves as well as increased plant growth, which suggests that nitrogen allocation to leaves improves carbon fixation and, vice versa, that nitrogen allocation to other parts of the plant than leaves, such as stem tissues, suppresses carbon fixation and growth of the plants. Based on these results, it seems possible that amino acid permeases, such as PotraAAP2.2, act to divert amino acids from the xylem sap, negatively influencing carbon fixation in the leaves and concomitantly carbon allocation to the stem and the radial growth. However, the expression of *PotraAAP2.2* in the xylem parenchyma as well as in the phloem (Figure 3, Figure S3 available as Supplementary Data at *Tree Physiology* Online) is different from the strictly phloem-specific expression of the Arabidopsis *AtAAP2* (Zhang et al. 2010). *PotraAAP2.2* expression is actually more similar to *AtAAP6* which is expressed in the xylem parenchyma cells (Okumoto et al. 2002). AtAAP6 has been proposed to have a role in xylem-to-phloem transport of amino acids (Okumoto et al. 2002), supporting function of PotraAAP2.2 in amino acid uptake from the sap. Future work is needed to clarify the function of these AAPs in amino acid reallocation in the stem and their effect on tree growth.

## Conclusions

**Figure 6:**
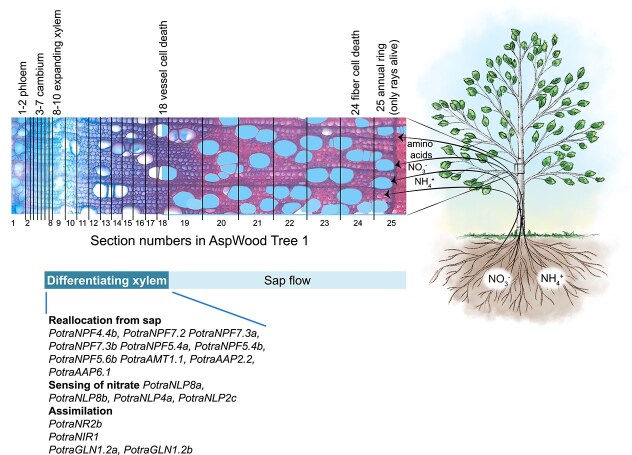
A proposed scheme of nitrogen uptake, reallocation and assimilation in the stem of aspen trees. The scheme is based on data presented on the expression of the aspen genes in AspWood (plantgenie.org) and gene co-expression patterns in the SwAsp population of aspen, presented in Figures 3 and 5. The microscopic image depicts the location of the tangential sections used for the gene expression analysis in the aspen stem in tree 1 in the AspWood data. The majority (>50%) of the cells in each section was estimated to reside in the phloem (sections 1 and 2), in the vascular cambium (sections 3–6), in the expanding xylem (sections 7–10) and in maturing xylem (the rest of the sections) according to Sundell et al. (2017). Vessel cell death was estimated to occur around the section number 18, and sap flow therefore takes place from sections 18–25. Fiber cell death was estimated to occur around section number 24. The only living cell type in section number 25 is therefore the rays. We provide evidence that both nitrate and ammonium is taken up by aspen roots at least in greenhouse conditions, and that each of nitrate, ammonium and amino acids are reallocated from the xylem sap to the parenchymatic ray cells and assimilated in the developing xylem tissues of the stem. Some of the proteins that seem to operate in nitrogen reallocation, nitrate sensing and nitrogen assimilation in the developing xylem, based on gene expression, are listed in the schematic representation.

We present a comprehensive analysis of nitrogen-related gene families in aspen, highlighting the genetic and functional dynamics essential for nitrogen-use efficiency. High-resolution gene expression analyses, together with co-expression analyses in a population of aspen trees, revealed a set of genes that are not typically associated with nitrogen uptake or assimilation and might therefore encode proteins that have specific functions in nitrogen reallocation and assimilation in the developing xylem tissues of the stem (Figure 6). The expression of these genes provide evidence on nitrate and ammonium uptake from the soil, uptake of nitrate, ammonium and amino acids from xylem sap into the parenchymatic ray cells, and assimilation in the developing xylem tissues of the stem (Figure 6).

We suggest on the basis of our gene expression analyses that even though nitrogen assimilation seems to be highly active in the developing xylem tissues of the stem, it needs to be suppressed by limiting the uptake of nitrate and amino acids from the xylem sap for the benefit of the photosynthetic nitrogen-use efficiency in the leaves. The negative correlation between the expression of specific members of the AAP and NRT1/NPF family members with plant diameter emphasizes their potential role in modulating nitrogen allocation, photosynthetic nitrogen-use efficiency and tree growth. Future investigations are warranted to validate these findings, paving the way for sustainable practices that optimize plant productivity in variable environmental conditions.

## Supplementary Material

Figure_S1_tpaf099

Figure_S2_tpaf099

Figure_S3_tpaf099

Table_S1_tpaf099

Table_S2_tpaf099

Table_S3_tpaf099

Supplementary_data_tpaf099

## Data Availability

The raw reads from RNA-Sequencing of ammonia treated hybrid aspen xylem are available under the NCBI project ID# PRJNA1169771. The script for DESeq analysis and heatmaps can be found at https://github.com/shruti281989/nitrogenDESeq.
