# Supplementary material for: Unraveling nitrogen uptake and metabolism: gene families, expression dynamics and functional insights in aspen (Populus tremula)
Source: Tree Physiol. 2025 Aug 11;45(13):100–13. doi: 10.1093/treephys/tpaf099 (PMC12666385; doi:10.1093/treephys/tpaf099)
Supplement: Figure_S1_tpaf099 [file figure_s1_tpaf099.pdf]

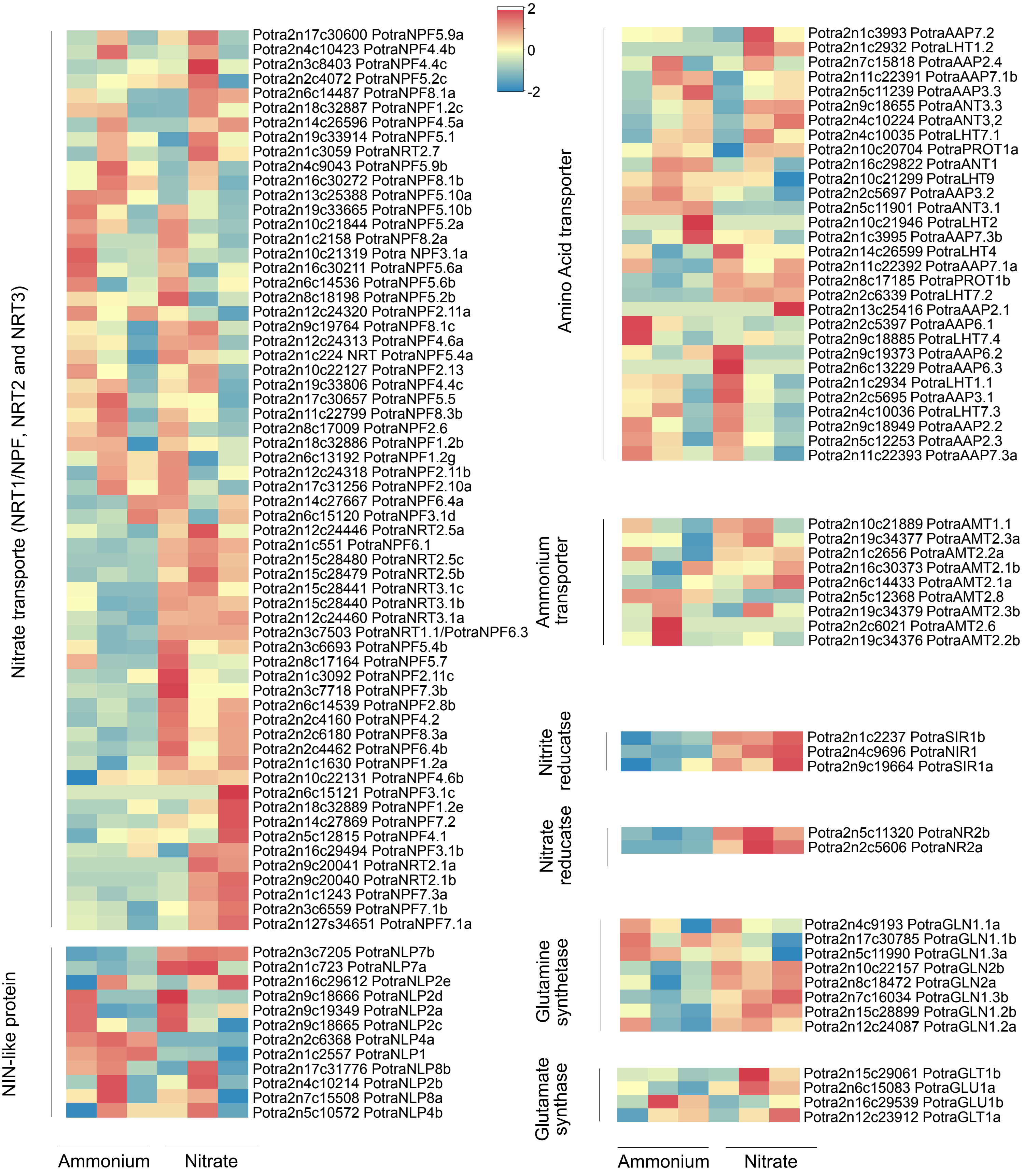

Figure S1. The expression of the members of *P. tremula* gene families related to nitrogen uptake, sensing and assimilation in response to fertilization with either nitrate or ammonium. The heatmaps represent gene expression data from RNA-sequencing of differentiating xylem tissues of hybrid aspen (*Populus tremula* × *P. tremuloides*) after long-term treatment with optimal, sub-optimal and limited doses of ammonia (NH_4^+, this study) and nitrate (NO_3^-, reported earlier in Renström et al. 2024). The data are VST normalized expression values scaled within each row (gene).
