# Supplementary material for: Unraveling nitrogen uptake and metabolism: gene families, expression dynamics and functional insights in aspen (Populus tremula)
Source: Tree Physiol. 2025 Aug 11;45(13):100–13. doi: 10.1093/treephys/tpaf099 (PMC12666385; doi:10.1093/treephys/tpaf099)
Supplement: Figure_S2_tpaf099 [file figure_s2_tpaf099.pdf]

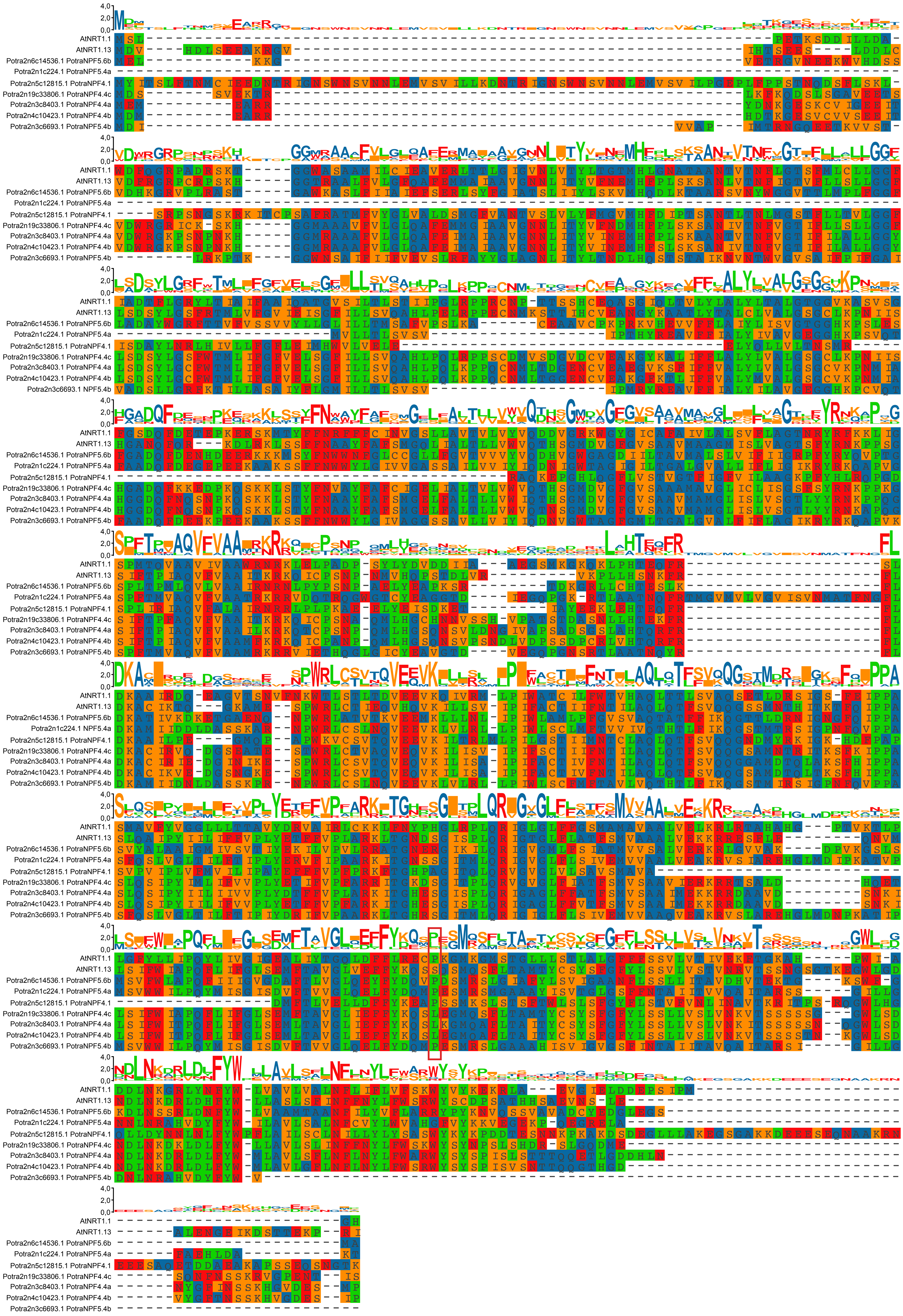

Figure S2. Presence of the proline residue in selected members of the NPF family in *Arabidopsis* and *Populus tremula*. The amino acid alignment was performed using the MAFFT. The aligned sequences were visualized by TBTOOL2. The red box indicates the proline residue in the aligned sequences.
