# Supplementary material for: Unraveling nitrogen uptake and metabolism: gene families, expression dynamics and functional insights in aspen (Populus tremula)
Source: Tree Physiol. 2025 Aug 11;45(13):100–13. doi: 10.1093/treephys/tpaf099 (PMC12666385; doi:10.1093/treephys/tpaf099)
Supplement: Figure_S3_tpaf099 [file figure_s3_tpaf099.pdf]

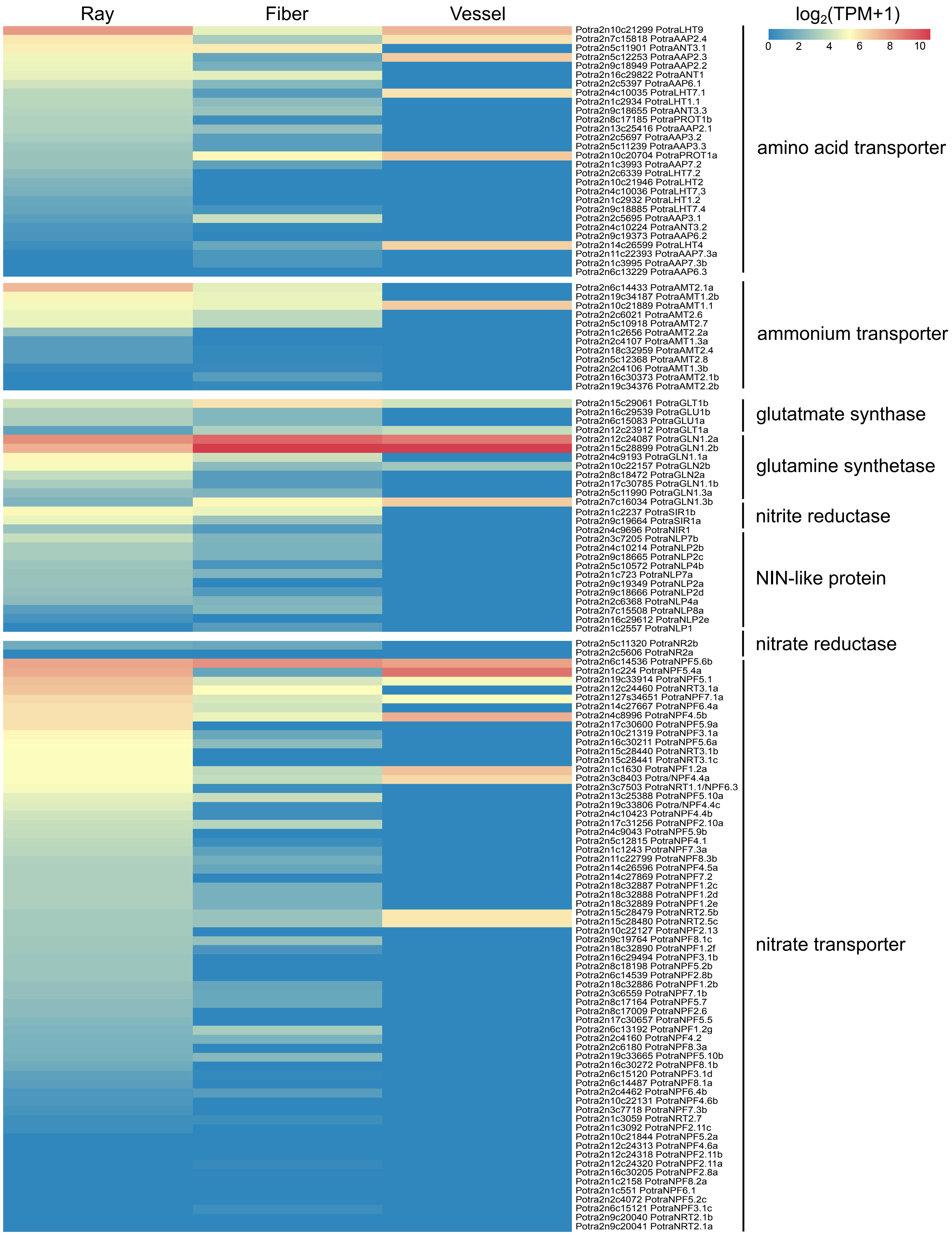

Figure S3. The expression of the members of the nitrogen metabolism related gene families in xylem rays, fibers, and vessels. The data is derived from RNA-sequencing of laser capture microdissected xylem cells from *P. trichocarpa*, performed by Tung et al. (2023). It should be noticed that the vessel sample (sample number 2 shown here) is likely to contain signal also from the other cell types, in particular the rays, as demonstrated in Tung et al. (2023). The gene expression data shown are log2(TPM+1) values
