# Supplementary material for: Unraveling nitrogen uptake and metabolism: gene families, expression dynamics and functional insights in aspen (Populus tremula)
Source: Tree Physiol. 2025 Aug 11;45(13):100–13. doi: 10.1093/treephys/tpaf099 (PMC12666385; doi:10.1093/treephys/tpaf099)
Supplement: Supplementary_data_tpaf099 [file supplementary_data_tpaf099.docx]

## Supplementary data

Figure S1. The expression of the members of aspen gene families related to nitrogen uptake, sensing and assimilation in response to fertilization with either nitrate or ammonium.

Figure S2. Presence of the proline residue in selected members of the NPF family in Arabidopsis and *Populus tremula*.

Figure S3. The expression of the members of the nitrogen uptake and metabolism related gene families in xylem rays, fibers, and vessels.

Table S1. Nitrogen uptake and metabolism related gene families in aspen (*Populus tremula)* and Arabidopsis.

Table S2. Gene expression analysis from RNA-sequencing of xylem tissue from hybrid aspen fertilized with either ammonium or nitrate.

Table S3. eQTL analysis of the NIN-like and NRT transcription factor family in a population of Swedish aspen trees.
